# On the Calculation of the Effective Polytropic Index in Space Plasmas

**DOI:** 10.3390/e21100997

**Published:** 2019-10-12

**Authors:** Georgios Nicolaou, George Livadiotis, Robert T. Wicks

**Affiliations:** 1 Department of Space and Climate Physics, Mullard Space Science Laboratory, University College London, Dorking, Surrey RH5 6NT, UK; r.wicks@ucl.ac.uk; 2 Southwest Research Institute, San Antonio, TX 78238, USA; george.livadiotis@swri.org

**Keywords:** polytropic index, space plasmas, solar wind

## Abstract

The polytropic index of space plasmas is typically determined from the relationship between the measured plasma density and temperature. In this study, we quantify the errors in the determination of the polytropic index, due to uncertainty in the analyzed measurements. We model the plasma density and temperature measurements for a certain polytropic index, and then, we apply the standard analysis to derive the polytropic index. We explore the accuracy of the derived polytropic index for a range of uncertainties in the modeled density and temperature and repeat for various polytropic indices. Our analysis shows that the uncertainties in the plasma density introduce a systematic error in the determination of the polytropic index which can lead to artificial isothermal relations, while the uncertainties in the plasma temperature increase the statistical error of the calculated polytropic index value. We analyze *Wind* spacecraft observations of the solar wind protons and we derive the polytropic index in selected intervals over 2002. The derived polytropic index is affected by the plasma measurement uncertainties, in a similar way as predicted by our model. Finally, we suggest a new data-analysis approach, based on a physical constraint, that reduces the amount of erroneous derivations.

## 1. Introduction

In a polytropic process, the plasma transits from one thermodynamic equilibrium state to another, under constant specific heat. The polytropic equation relates the plasma density *n* and temperature *T* or pressure *P* (e.g., [1,2]):

(1) $$P\propto n^{\gamma }\;\mathrm{or}\;T\propto n^{\gamma -1},$$

where *γ* is the plasma polytropic index. The polytropic relationship is valid within specific plasma parcels, therefore should be examined within a specific family of streamlines of the solar wind flow. The value of the polytropic index is characteristic of the type of the process the plasma experiences. For *γ* = ∞ the process is isochoric, and the plasma density does not change during the transition from one state to another. For *γ* = 5/3, there is no heat transfer during the plasma expansion or compression, and the process is called adiabatic. For *γ* = 1, the process is isothermal (constant temperature) and for *γ* = 0 is isobaric (constant pressure). Finally, a negative polytropic index characterizes plasma explosive behavior. There are also cases where the plasma is not characterized by a single polytropic index. In this consideration, the plasma may reside in a superposition of states, each described by a single polytropic index [3]. Another possibility is to have a non-homogeneous polytropic index [4].

The accurate determination of the polytropic index in plasmas is of crucial importance since it is directly related to the thermodynamic state of the plasma. It is a key parameter in the equation of state and it is directly related to the effective degrees of freedom characterizing various dynamical processes. Gas dynamic and magneto hydro dynamic (MHD) models require the polytropic index, thus, a realistic value is crucial for the correct interpretation of the results.

For instance, the knowledge of the polytropic index is needed for the accurate description of the ambient solar wind expansion (e.g., [5]), the plasma dynamics within large scale structures such as magnetic clouds (e.g., [6,7]), planetary magnetospheres (e.g., [8]), and the inner heliosheath (e.g., [9]). Additionally, the polytropic index defines the boundary conditions across discontinuities (e.g., [4,10,11]). Plasma modeling is also essential to some recent high-profile astrophysics results, e.g., the first image of a black hole is modeled using MHD with a polytropic index [12]. Measurements in the solar wind and lessons learned from the more constrained modeling of the solar wind can be applied to these systems.

The accurate determination of *γ* leads also to the accurate description of the plasma parameters in small scale structures, such as turbulent solar wind fluctuations (e.g., [13] and references therein). The polytropic relationship is directly related to plasma thermodynamics as it determines the amount of heat that is supplied or emitted from the plasma during a specific process. Recently, references [14,15,16], showed that *γ* is directly related to the distribution function of the plasma particle velocities and the potential energy in the plasma, concluding that *γ* becomes an essential parameter for the complete study of the plasma dynamics.

In space plasma applications, the polytropic index can be determined from the linear relation between ln(*n*) and ln(*T*) along individual streamlines:

(2) lnT=(γ−1)lnn+const.

For example, Newbury et al. [17], examined the relation between the plasma density and temperature in the vicinity of stream interactions, using observations by Pioneer Venus Orbiter (PVO). Their analysis revealed streams with adiabatic behavior, and a few streams with *γ* ~ 2, which indicates that the degrees of freedom are occasionally restricted.

Kartalev et al. [18], proposed an approach for detecting intervals corresponding to plasma within the same streamline and used a linear regression model between the logarithms of plasma density and temperature to derive *γ*. The authors of this paper applied their method to derive the polytropic index of the plasma within a magnetic cloud observed by Wind. The analysis determined *γ* < 1, which was previously suggested by Osherovic et al. [19] for ideal, axially symmetric, magnetic clouds. The specific result indicates that as the magnetic cloud expands, heat is supplied to the system and the temperature increases.

Pang et al. [20], analyzed Cluster observations and derived the polytropic index of the terrestrial magnetosheath ions. Following the approach by Kartalev et al. [18], they analyzed intervals for which the Bernoulli integral is quasi-constant. For each selected (suitable) interval, they derived *γ* by determining the linear relation between ln(*P*) and ln(*n*). They determined polytropic indices within the range from −2 to 3, with median value ~1. Park et al. [21] performed a statistical study of the polytropic index of the terrestrial magnetosheath ions, over a 10-year period using THEMIS observations. The authors concluded that *γ* depends on the bow shock geometry and it is presumably controlled by turbulence, waves, and instabilities (e.g., [22,23]).

In Nicolaou et al. [24], we analyzed 1-min resolution measurements of the solar wind proton density and temperature from 1995 to 2012. We examined the polytropic relation within 8-min time intervals, by applying the linear model of Equation (2) to the data within each interval. We determined the distribution of *γ* and calculated an average value of *γ* ~1.8. Among others, we demonstrated that large uncertainties in the plasma density can lead to poorly fitted data, and the traditional fitting in the log-log scale will determine slopes ~0, which could be misinterpreted as artificial isothermal cases with *γ* = 1. Therefore, we filtered the derived polytropic indices according to the correlation coefficient characterizing the linear fit to the data. This specific filter removes not only the poorly fitted data, but also the real quasi-isothermal cases (e.g., Figure 3 in Nicolaou et al. [24]).

Similar techniques were used in [25] to analyze solar wind proton plasma measurements by Wind in selected time intervals during the first 70 days of 1995, and the mean value of ~1.6–1.8 was derived for *γ*. More recently, Livadiotis [26], used Wind data to calculate the polytropic index of solar wind protons in selected streamlines over the last two solar cycles. The study determined an average *γ* ~1.8, confirming the result by Nicolaou et al. [24]. Additionally, the polytropic index is found to have no dependence on solar wind speed. This is usually used to characterize the state of the solar wind [27]. The specific result confirms the earlier study by Totten et al. [5] who determined *γ* based on the radial profiles of *n* and *T* as measured by Helios.

Several other studies used the linear fit method to derive the polytropic indices of different plasma species in several plasma regimes. For example, Dialynas et al. [28], examined the polytropic index of energetic H^+^ and O^+^ in Saturn’s magnetosphere, revealing a sub-adiabatic behavior on average. Similarly, Arridge et al. [29] calculated the polytropic index of electrons in the Saturnian magnetotail and determined a quasi-isothermal behavior. Nicolaou et al. [30] attempted to determine the polytropic index in the distant Jovian magnetosheath protons using data by New Horizons, and they derived *γ* ~0. The polytropic index of ions in the heliosheath is also calculated close to *γ* ~0, indicating an isobaric behavior (e.g., [31,32,33]).

Although all the studies mentioned above successfully derived the polytropic index of the plasma in different environments, as far as we are aware, there is no dedicated study that quantifies the systematic and statistical errors of *γ* as a function of the measurement uncertainties. In this paper, we investigate the impact of uncertainties of thermal observables on the estimation of the polytropic index. In particular, we quantify the effects of the uncertainties in the plasma density and temperature on the determination of *γ* using the traditional linear fitting of ln*T* as a function of ln*n* in Equation (2). We model plasma observations for a range of polytropic indices, assuming specific uncertainty in the measurements. Then, we analyze the modeled observations with the standard technique to derive the polytropic index *γ*, which we then compare with the input value we use to model the observations. The comparison between the derived and the input values of *γ* quantifies the error of *γ* as a function of the input uncertainty in the plasma measurements. Such quantification can indicate the erroneous data which can then be removed from future data-analyses.

In the next section, we describe in detail our model for the plasma density *n* and temperature *T* measurements and their uncertainties. In Section 3 we describe our analysis techniques, which we use to derive the polytropic indices from the modeled plasma density and temperature measurements. In Section 4, we show the results of our model and we discuss the predicted misestimation of *γ* as a function of the relative errors $\sigma_{n}/n$ and $\sigma_{T}/T$. In Section 5, we analyze Wind observations of solar wind protons near 1 au, and we compare the predictions of our model to the examined datasets. In Section 6, we discuss our results, and we suggest a new analysis approach to overcome the exposed misestimation. Finally, Section 7 summarizes our key findings and conclusions.

## 2. Model

### 2.1. Density and Temperature Data-Points

**Figure 1:**
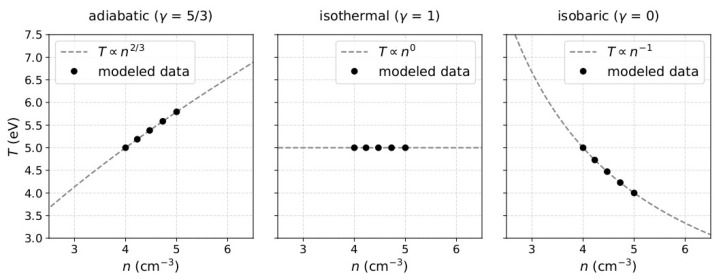
Model of plasma temperature *T* as a function of the modeled plasma density *n* for (**left**) adiabatic plasma with *γ* = 5/3, (**middle**) isothermal plasma with *γ* = 1 and (**right**) for isobaric plasma with *γ* = 0. In each panel, we plot $T\propto n^{\gamma -1}$ (grey dashed). For all the examples here, the plasma density ranges between *n*_min_ = 4 cm^−3^ and *n*_max_ = 5 cm^−3^ and *T*_0_ = 5 eV.

We simulate the plasma density and temperature for specific polytropes, characterized by a polytropic index *γ*. For each *γ* value, we consider five consecutive values of the plasma density *n_i_*, logarithmically spread over the range Δ*n* = *n*_max_ − *n*_min_. For each *n_i_* point we model a plasma temperature value *T*_i_ according to the polytropic relation:

(3) $$T_{i}=Cn_{i}{}^{\gamma -1},$$

with the constant

(4) $$C=T_{0}n_{\min}{}^{1-\gamma },$$

which we define for the minimum value of density *n*_min_ and a corresponding reference temperature value *T*_0_ within the interval. Note that, for *γ* > 1, *T*_0_ is the minimum temperature *T*_min_, while for *γ* < 1, is the maximum temperature *T*_max_ within the time interval. The number of data-points, *n*_min_, *n*_max_, *T*_min_, and *T*_max_ within each modeled interval should be adjusted for specific applications, as we do in Section 4 and Section 5. However, for the general model demonstration here, we consider typical values of solar wind plasma protons, within short time intervals (up to eight minutes) covered in five consecutive observations of ~1 to 1.5-min resolution (e.g., [14,16,24,25,26]). The selection of short time intervals in the referred studies, aims to reduce the possibility of streamline mixing within the observations. In Figure 1, we show examples of the modeled density and temperature for three characteristic values of *γ*.

### 2.2. Density and Temperature Uncertainties

The uncertainties (one-sigma error) of the observed density and temperature are

(5) $$\sigma_{n}=\sqrt{{\sigma_{n,f}}^{2}+{\sigma_{n,m}}^{2}}\;\mathrm{and}\;\sigma_{T}=\sqrt{{\sigma_{T,f}}^{2}+{\sigma_{T,m}}^{2}},$$

respectively. The subscript f denotes the uncertainties of the parameters due to natural plasma fluctuations (e.g., shocks, turbulence) in time scales shorter than the time resolution of the measurements. Typical plasma instruments complete a measurement by sampling the plasma in a finite time, and changes in the plasma parameters within the sampling time are not resolved [34]. These uncertainties are dependent on the polytropic relation. The subscript m denotes the measurement uncertainties of the quantities due to limited counting statistics and instrument capabilities (e.g., limited angular and energy resolution). Typical solar wind proton measurement uncertainties are within a few percent (e.g., [35]). Moreover, for high time resolution measurements, we can assume that $\sigma_{n,f}\ll \sigma_{n,m}$ and $\sigma_{T,f}\ll \sigma_{T,m}$, therefore,

(6) $$\sigma_{n}\approx \sigma_{n,m}\;\mathrm{and}\;\sigma_{T}\approx \sigma_{T,m}.$$

**Figure 2:**
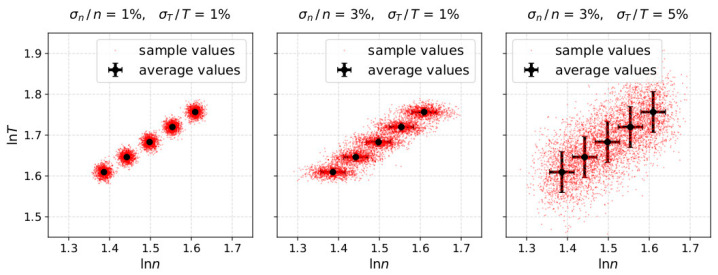
Modeled samples of ln*T* as a function of ln*n*, for adiabatic plasma and (**left**) $\sigma_{n}/n$ = $\sigma_{T}/T$ = 1%, (**middle**) $\sigma_{n}/n$ = 3%, $\sigma_{T}/T$ = 1% and (**right**) $\sigma_{n}/n$ = 3%, $\sigma_{T}/T$ = 5%. In each plot, the black data-points correspond to the modeled plasma parameters while the red dots correspond to the measurement samples. We model 1000 measurement samples, considering a log-normal distribution around the plasma parameters (black dots) and standard deviation as indicated by the error bar.

This work quantifies the misestimation of *γ* due to *σ_n_* and *σ_T_*. For the purpose of our study, we assign uncertainty in the modeled *n_i_* and *T_i_* by modeling 1000 measurement samples of each data-point, assuming that the 1000 samples, follow a log-normal distribution (e.g., [35,36,37]) with standard deviation determined by the uncertainty level. We specifically model $n_{ij}=e^{\ln n_{ij}}$ and $T_{ij}=e^{\ln T_{ij}}$ with the index *i* denoting the data-point within the interval (ranging from 1 to 5) and *j* denoting the measurement sample (ranging from 1 to 1000). The 1000 $\ln n_{ij}$ and $\ln T_{ij}$ values of each *i*th data-point, are normally distributed, with mean values $\ln n_{i}$ and $\ln T_{i}$ following the polytropic model as described above, and non-dependent standard deviations $\sigma_{\ln n_{i}}={\sigma_{ni}/n}_{i}$ and $\sigma_{\ln T_{i}}=\sigma_{Ti}/T_{i}$, respectively. In Figure 2 we show three examples of modeled samples with different uncertainty in the plasma density and temperature.

## 3. Analysis

**Figure 3:**
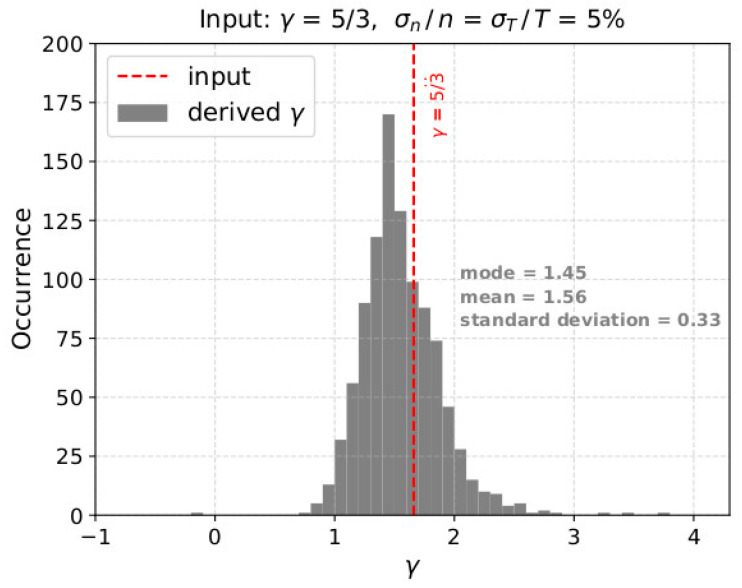
Histogram of the derived *γ* over 1000 samples, considering adiabatic plasma, and uncertainties $\sigma_{n}/n$ = $\sigma_{T}/T$ = 5%. Although the modeled plasma has *γ* = 5/3, the distribution of the derived values is slightly asymmetric with the most frequent value 1.45 and mean *γ*_m_ ~1.56. The standard deviation of the distribution is *σ_γ_* ~0.33. The plasma uncertainty in the plasma parameters introduces a systematical (different mean and mode) and statistical (*σ_γ_* > 0) error in the calculation of *γ*.

As described above, for each input polytropic index *γ* value and uncertainty level, we model 1000 interval samples. We then use a traditional chi-squared minimization method to fit the linear model of Equation (2) to the modeled ln*T* as a function of the modeled ln*n*, in each of the 1000 samples. Therefore, for each input, we derive 1000 values of *γ* using a fitting analysis which is used for actual space plasma applications. In Figure 3, we show the histogram of the 1000 polytropic index values as derived from the analysis of an adiabatic plasma model with density and temperature uncertainty σ*_n/_n* = σ*_T/_T* = 5%. As we introduce uncertainty in the plasma measurements, *γ* is derived within a finite range of values. In addition, the average and the most frequent values of the derived *γ* are different from the input value. The comparison between the input and the derived *γ* values, allows the quantification of the analysis accuracy as a function of the density and temperature uncertainties.

## 4. Results

**Figure 4:**
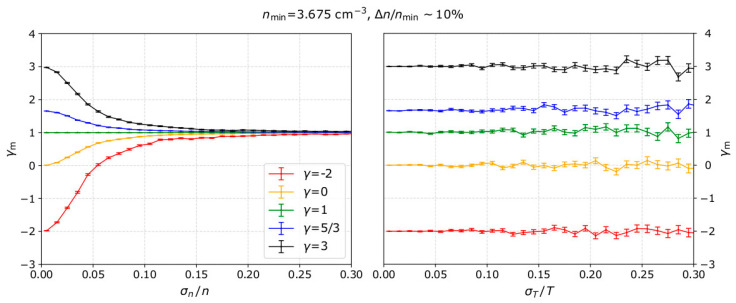
The average *γ*_m_ as a function of (**left**) density and (**right**) temperature measurement uncertainty. The average *γ*_m_ and its standard error δ*_γ_* are calculated over 1000 samples of *n*-*T* measurements. Lines with different colors represent different input *γ* values. For the specific examples, we set *n*_min_ = 3.675 cm^−3^, Δ*n* = n_max_ − n_min_ = 0.35 cm^−3^, and *T*_0_ = 3.275 eV, which correspond to typical values of solar wind protons as observed by Wind in 2002.

For each input *γ*, and specific uncertainty level, we calculate the mean *γ*_m_ and the standard error of the mean δ*_γ_* as calculated for 1000 modeled samples. We then repeat for several uncertainty levels and for different input *γ* values. In the left panel of Figure 4, we show the calculated mean *γ*_m_ as a function of the uncertainty in the plasma density measurements and for no uncertainty in the temperature measurements, while the right panel shows the results as a function of the temperature measurement uncertainty and for no uncertainty in the density measurements. Both panels show the results for five different input *γ* values. For all the examples in Figure 4 we set *n*_min_ = 3.675 cm^−3^, Δ*n* = *n*_max_ − *n*_min_ = 0.35 cm^−3^, and *T*_0_ = 3.275 eV, which are the most frequent values for solar wind plasma protons measured by Wind in 2002 (see Section 5).

Our results show that, with the standard linear fitting of ln*T* as a function of ln*n*, the polytropic index is misestimated due to uncertainties in the plasma density. As $\sigma_{n}/n$ increases, the mean *γ*_m_ is shifted towards 1. For example, when analyzing plasma with polytropic index *γ* = 3 (black line in Figure 4), the standard fitting analysis will estimate *γ* < 1.5 as $\sigma_{n}/n$ > 10% within the analyzed intervals. Additionally, for all the *γ* values we examine here, the plasma is misinterpreted as nearly-isothermal (*γ*~1) for $\sigma_{n}/n$ > 15%.

The right panel of Figure 4 indicates that there is no systematic misestimation of the polytropic index as a function of the temperature uncertainties. On the other hand, the standard error of the mean polytropic index δ*_γ_*, increases with $\sigma_{T}/T$, which means that the polytropic index is calculated within a broader range of values. For all the examples we examine here, the standard error δ*_γ_* does not exceed a few percent of the mean value *γ*_m_.

**Figure 5:**
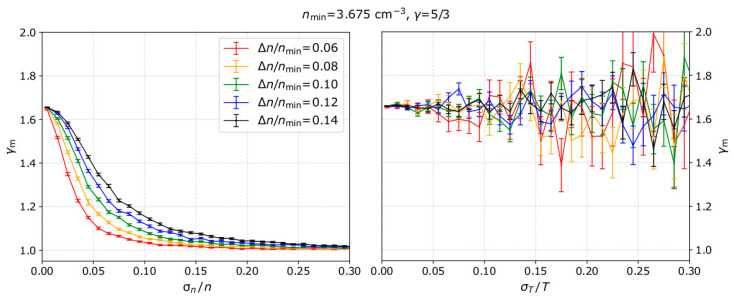
The derived polytropic index averages (over 1000 samples) as a function of (**left**) density and (**right**) temperature measurement uncertainty, for several Δ*n* ranges. For all the examples shown here, we consider an adiabatic plasma (input *γ* = 5/3).

We further examine the dependence of the derived *γ*_m_ on $\sigma_{n}/n$ and $\sigma_{T}/T$, for several Δ*n* ranges within the analyzed subintervals. In Figure 5, we show our model results for adiabatic plasma and Δ*n*/*n*_min_ values ranging from 0.06 to 0.14. The linear fitting is more accurate as Δ*n*/*n*_min_ increases. In other words, for the same level of plasma uncertainties, the accuracy of the fit increases as the actual change of the plasma density within the interval increases. Additionally, for the range of plasma parameters we examine here, the uncertainty in the plasma temperature introduces noise in the derived *γ*.

## 5. Application to Solar Wind Protons

**Figure 6:**
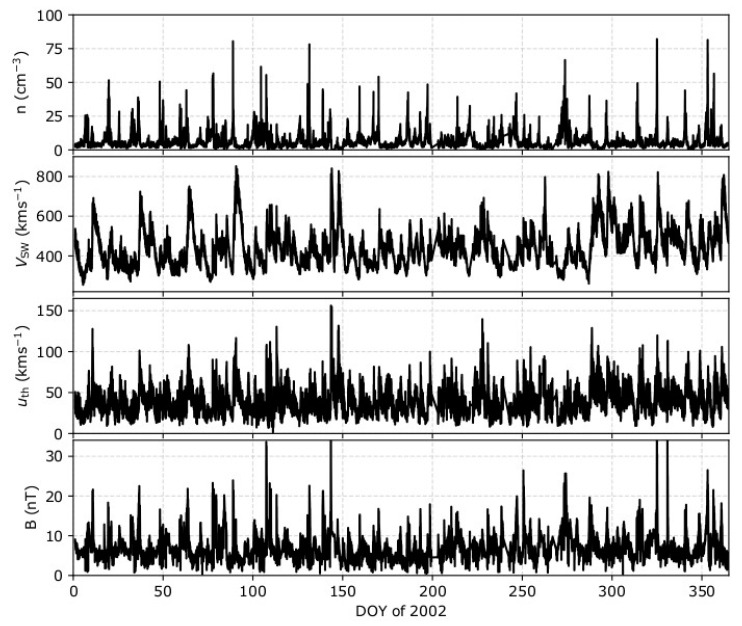
Wind high-resolution measurements of (from top to bottom) density, bulk speed, thermal speed, and magnetic field strength, during 2002.

Our model predicts that uncertainties in the plasma measurements can lead to significant misestimation of the polytropic index. We now investigate and apply the model predictions to solar wind data. We analyze high-resolution (92 s) solar wind proton and magnetic field observations by Wind spacecraft. Wind’s Solar Wind Experiment (Wind/SWE, [38]) measures the reduced velocity distribution of solar wind protons, from which proton bulk parameters are derived by non-linear fitting of bi-Maxwellian distribution functions to the observations [39]. The non-linear fitting also provides the one-sigma error of the derived parameters. The solar wind magnetic field components, which we use to estimate the Bernoulli integral within selected intervals, are measured by Wind/MFI [40]. Here, we analyze the data obtained in 2002. The specific year does not have large data-gabs, and moreover, it has a significant amount of suitable intervals (~250,000) for a polytropic index analysis as a function of the parameter uncertainties. Figure 6 shows the time series of the plasma density *n*, bulk speed *V*_sw_, thermal speed *u*_th_, and magnetic field *B*, as determined from non-linear fitting to the plasma observations, and the magnetic field magnitude, over the year 2002. (All datasets can be found in: https://cdaweb.gsfc.nasa.gov/index.html/).

**Figure 7:**
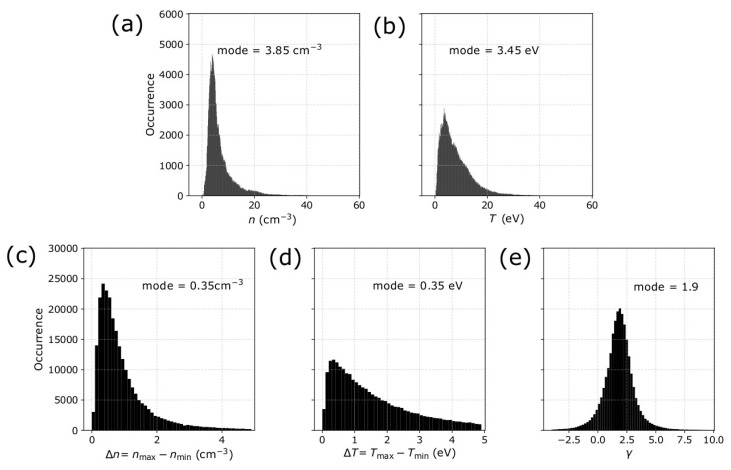
Histogram of the average solar wind protons parameters within the selected subintervals in 2002, which we analyze to derive the polytropic index, (**a**) the average density *n* and (**b**) average temperature *T*, (**c**) density range Δ*n*, (**d**) temperature range Δ*T* and (**e**) the derived polytropic index *γ*. In each panel, we note the most frequent value (mode) of each parameter, which we use as input to our model to predict the misestimation of *γ* as a function of the measurement uncertainties.

Following the analyses by references [3,14,16,18,20,24,26], we select intervals of five consecutive measurements (covering ~8 min of observations) that are characterized by a quasi-constant Bernoulli integral, and we derive *γ* by applying a linear fitting to ln*T* as a function of ln*n*. In Figure 7, we show the histogram of the average *n*, *T*, Δ*n*, Δ*T*, and *γ* within the analyzed intervals in 2002. In each panel, we indicate the most frequent value (mode) of the corresponding parameters.

**Figure 8:**
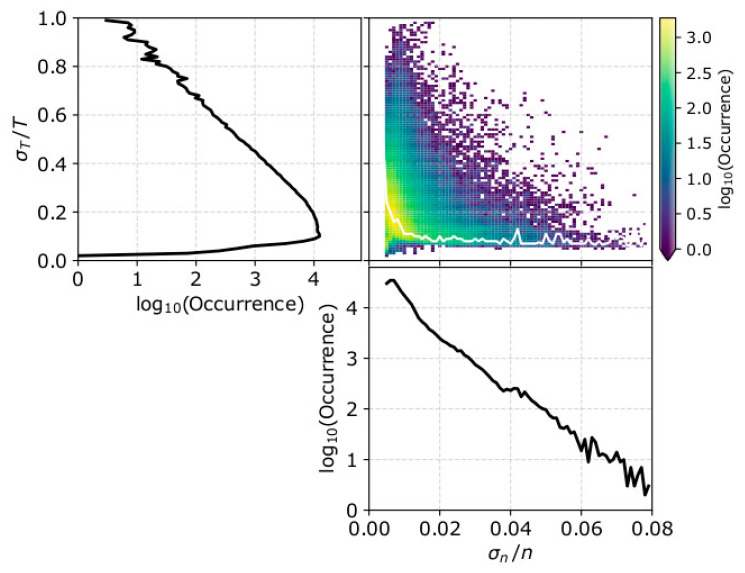
Occurrence of (**upper left**) the average $\sigma_{T}/T$, (**lower**) the average $\sigma_{n}/n$ and (**upper right**) the 2D histogram of $\sigma_{T}/T$ and $\sigma_{n}/n$ of the Wind observations in 2002. The white line indicates the mode of $\sigma_{T}/T$ in each $\sigma_{n}/n$ bin.

We also calculate the average (over the five consecutive measurements) sigma error $\sigma_{n}/n$ and $\sigma_{T}/T$ within each subinterval. Figure 8 shows the occurrences of $\sigma_{T}/T$, $\sigma_{n}/n$ and their 2D histogram for the analyzed subintervals in 2002. The relative density error $\sigma_{n}/n$ is recorded up to ~8% while the temperature relative error $\sigma_{T}/T$ extends up to ~100%. We remind the reader that for a straight comparison with our model, we assume that the relative errors $\sigma_{n}/n$ and $\sigma_{T}/T$ are fair estimates of $\sigma_{\ln n}$ and $\sigma_{\ln T}$, the standard deviations of ln*n* and ln*T,* respectively. The 2D histogram in the top right panel indicates that there is an anti-correlation between $\sigma_{n}/n$ and $\sigma_{T}/T$. In order to compare the effects of one parameter uncertainty on the calculation of *γ*, we need to eliminate the effect of the uncertainty in the other parameter. Therefore, we select threshold values of $\sigma_{n}/n$ < 1% and $\sigma_{T}/T$ < 15%, chosen so that while we eliminate the effects of the corresponding parameter, we include a significant amount of data-points over a wide range of the examined parameters (see Figure 8).

**Figure 9:**
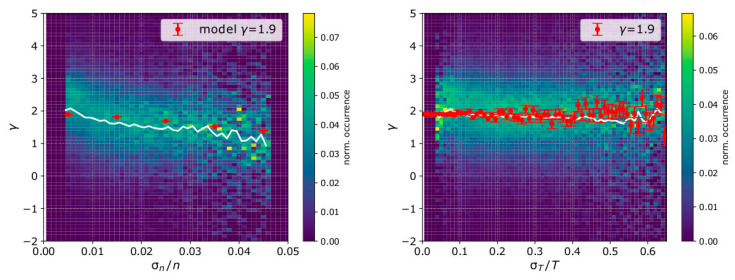
Normalized histograms of (**left**) *γ* as a function of $\sigma_{n}/n$, for $\sigma_{T}/T$ < 15% and (**right**) *γ* as a function of $\sigma_{T}/T$, for $\sigma_{n}/n$ < 1%. The white line is the mean value of the histogram in each column. We display only the range of uncertainties for which we have more than 100 data points. On each panel, we show the predictions of our model (red) for plasma parameters corresponding to the mode values of each parameter for the analyzed intervals (see also Figure 7).

We further examine the calculated polytropic index as a function of the measurement uncertainties. The left panel of Figure 9 shows the 2D histogram of *γ* occurrence (normalized per column) as a function of $\sigma_{n}/n$, for $\sigma_{T}/T$ < 15%, while the right panel shows the corresponding 2D histogram of *γ* as a function of $\sigma_{T}/T$, for $\sigma_{n}/n$ < 1%. On the top of each histogram, we plot the predictions of our model for input parameters *n*, *T*, Δ*n*, Δ*T,* and *γ*, set to the mode values of the corresponding parameters within the analyzed intervals over 2002 (mode values shown in Figure 7).

The data-analysis of the solar wind protons shows a clear dependence of the calculated *γ* as a function of $\sigma_{n}/n$. For low levels of density uncertainty, the average *γ* ~ 1.9, while it drops to *γ* ~ 1.5 as σ*_n/_n* ~ 5%. Our model suggests that while the actual average *γ* ~1.9, it is possibly underestimated as the density measurement uncertainty increases. The uncertainty in the plasma temperature does not cause any systematic misestimation of *γ*, which is also compatible with our model predictions. Although our model considers one universal value for *γ*, the actual measurements possibly include plasma structures that expand under different conditions, which contributes to the spread of the calculated *γ* in Figure 9. However, we consider that our model values can represent the mean values of the measured *γ*, and we argue that the systematic behavior of *γ* as a function of the uncertainties could be an artifact related to the fitting method and not necessarily an actual difference in the plasma expansion conditions. We discuss our results further, in the next section.

## 6. Discussion

We show that the traditional calculation of the polytropic index from the linear fitting to ln*T* as a function of ln*n* data is sensitive to the accuracy with which the plasma parameters are measured. The uncertainties in the plasma density introduce random scattering of the fitted data-points along the x-direction of the Cartesian coordinate system we use for the fitting. As the scattering increases, the chi-squared value of the fitting is minimized for a linear model with slope closer to 0, introducing systematic error in the calculation of *γ* and occasionally, lead to artificial isothermal indices (*γ* ~ 1). On the other hand, uncertainties in the plasma temperature introduce random scattering of the data-points along the y-direction, leading to a larger statistical error in the calculation of *γ*. A relevant study by Livadiotis [41] investigates and discusses in detail the accuracy of the linear fitting method as a function of the reference frame. The magnitude of the propagated error (systematic and statistical) in *γ* is a function of the relative errors, $\sigma_{n}/n$ and $\sigma_{T}/T$, and the corresponding parameter ranges Δ*n* = *n*_max_ − *n*_min_ and Δ*T* = *T*_max_ − *T*_min_ in the examined intervals. We also expose this misestimation by analyzing actual solar wind proton data. Here, we discuss possible ways to filter the data and avoid erroneous calculations of *γ*.

### 6.1. Uncertainty Threasholds

One reasonable and straight forward approach that reduces the error in the calculated *γ*, is the data filtering, which excludes data-points with large measurement uncertainties. Although such an approach reduces the amount of analyzed data, it will improve the accuracy of the derived *γ* indices. Our study quantifies the linear fit accuracy as a function of the measurement uncertainties, the range of *n* within the intervals, and for several *γ* indices (e.g., Figure 4 and Figure 5). In conclusion, future polytropic index analyses that use the traditional fitting should follow our techniques to optimize the thresholds in their data-filtering according to the accuracy requirement in each application.

### 6.2. Correlation Coefficient Filter

**Figure 10:**
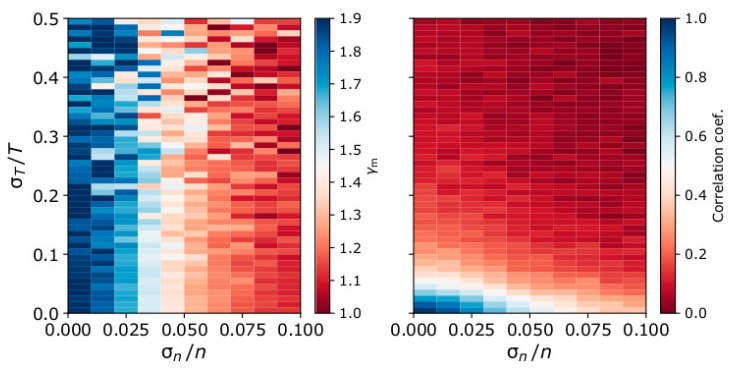
The 2D histogram of (**left**) mean calculated polytropic index *γ*_m_ and (**right**) the average Pearson correlation coefficient of ln*T* and ln*n* as a function of $\sigma_{n}/n$ and $\sigma_{T}/T$ for modeled plasma with *γ* = 1.9, *n*_min_ = 3.675 cm^−3^, Δ*n* = n_max_ − n_min_ = 0.35 cm^−3^, and *T*_0_ = 3.275 eV. The average polytropic indices and correlation coefficients are calculated for 1000 modeled measurement samples per each combination of $\sigma_{n}/n$ − $\sigma_{T}/T$.

Large uncertainties in the plasma density and temperature reduce the absolute correlation coefficient value of the linear fits to the ln*T*–ln*n* data. In the left panel of Figure 10, we plot the 2D histogram of the mean *γ*_m_ as a function of $\sigma_{n}/n$ and $\sigma_{T}/T$ as predicted by our model for input *γ* = 1.9. In the right panel of Figure 10, we show the 2D histograms of the average correlation coefficient of the linear fittings to ln*T*–ln*n*. The average *γ*_m_ and the average correlation coefficients are calculated over 1000 modeled samples for each $\sigma_{n}/n$ and $\sigma_{T}/T$ combination. One approach to reducing the amount of erroneous data is to filter the results based on the correlation coefficient between ln*T*–ln*n* within the analyzed intervals. The selected correlation threshold should be optimized according to the desired accuracy. Nevertheless, such a filter excludes real isothermal cases from the analysis (e.g., [24]).

### 6.3. Proposed Approach Cased on the Special Polytropic Index V

According to the polytropic relation in Equation (1), the density as a function of temperature is

(7) $$n\propto T^{1/\left(\gamma -1\right)}\;\mathrm{or}\;n\propto T^{\nu },$$

where the special polytropic index

(8) $$\nu \equiv {\left(\gamma -1\right)}^{-1}.$$

Taking the logarithm of Equation (7):

(9) lnn=νlnT+const.,

and in data-analyses, the index *ν* can be calculated by applying a linear model to ln*n* as a function of ln*T*. The function in Equation (9) is the inverse of the function in Equation (2), and the derived by a fitting index *ν*_inv_, similarly to *γ*, is also affected by the plasma measurement uncertainties.

**Figure 11:**
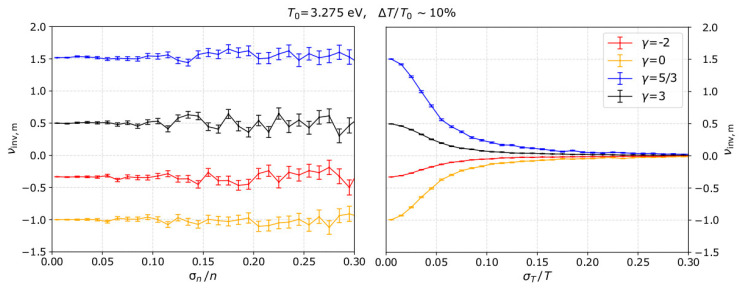
(**Left**) average *ν*_inv_,_m_ as a function of $\sigma_{n}/n$ for $\sigma_{T}/T$ = 0 and (**right**) as a function of $\sigma_{T}/T$ for $\sigma_{n}/n$ = 0 and for several *γ*. The average *ν*_inv,m_ is calculated over 1000 samples for each uncertainty setting in our model. For the specific examples we consider *T*_0_ = 3.275 eV and $\Delta T/T_{0}$ ~ 10%.

In order to demonstrate and examine the dependence of *ν*_inv_ on the measurement uncertainties, we run model plasma measurements in a similar manner as described in Section 2, and we derive *ν*_inv_ as the slope of the line fitted to ln*n* as a function of ln*T*. In the left panel of Figure 11, we plot the average estimated *ν*_inv,m_ as a function of $\sigma_{n}/n$ for $\sigma_{T}/T$ = 0, while in the right panel we show *ν*_inv_,_m_ as a function of $\sigma_{T}/T$ for $\sigma_{n}/n$ = 0. In such a chi-square minimization fitting, the uncertainties in the plasma density propagate a statistical error in the calculation of *ν*_inv_, while uncertainties in the plasma temperature introduce a systematical error by shifting the estimated *ν*_inv_ towards zero.

The accuracy of the estimated *γ* and *ν*_inv_, is affected by $\sigma_{n}/n$ and $\sigma_{T}/T$. However, (*γ*-1) and *ν*_inv_ are the slopes of two inverse functions and are related by the identity in Equation (8) which we rewrite as

(10) $$\nu_{\mathrm{inv}}(\gamma -1)=1\Rightarrow \frac{1}{\nu_{\mathrm{inv}}}-(\gamma -1)=0.$$

**Figure 12:**
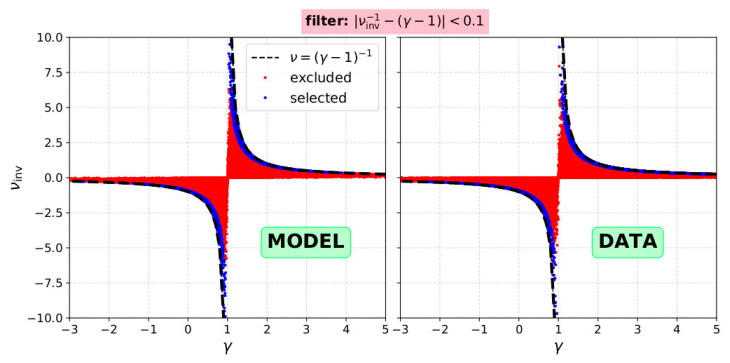
(**Left**) the calculated *ν* as a function of the calculated *γ* for 250,000 samples with input *γ* = 1.9, $\sigma_{n}/n$ = $\sigma_{T}/T$ = 8%, and (**right**) the same plot for the analyzed intervals from Wind measurements during 2002. In both plots, the blue data-points satisfy the criterion | 1/*ν*_inv_ – (*γ*-1)| < 0.1, while the red-data points do not, as they lie further from the expected *ν* ≡ (*γ* − 1)^−1^ (dashed).

Equation (10) implies that we need to validate intervals for which

(11) $$\left|\frac{1}{\nu_{\mathrm{inv}}}-(\gamma -1)\right|<\alpha ,$$

where *α* is a threshold value to be determined according to the desired accuracy of the results. In Figure 12 we plot the calculated *ν* as a function of the calculated *γ* for ~250,000 samples (comparable to the amount of analyzed intervals in 2002) with input *γ* = 1.9 and $\sigma_{n}/n$ = $\sigma_{T}/T$ = 8%. The input uncertainty in the parameters considers the highest levels in density uncertainty measured in 2002 (see Figure 8). We demonstrate which data-points will be excluded as we apply a filter according to Equation (11) for a threshold value *α* = 0.1. In the right panel of Figure 12, we show in the same format, the indices as calculated form Wind data during 2002. In both plots, the dashed line indicates the ν ≡ (*γ* − 1)^−1^. The distribution of the data points in the model and the distribution of the actual data are virtually the same. We emphasize on the fact that as expected, for *ν* > 0, all the data-points reside on the left of the dashed line, and for *ν* < 0, all the data-points reside on the right of the dashed line. This reflects the fact that the uncertainties in *n* shift *γ* towards 1, while uncertainties in *T* shift *ν*_inv_ towards 0.

**Figure 13:**
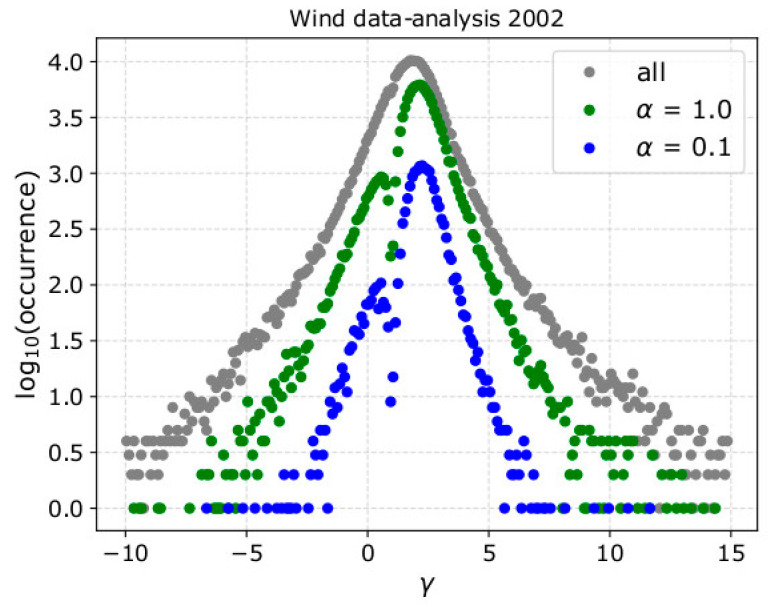
Histograms of *γ* as derived by the analysis of Wind observation over 2002, before (grey) and after the filter application with (green) α = 1, and (blue) α = 0.1. The filtered *γ* values are recorded within a shorter range, and the corresponding histogram has a sharp dip at *γ* = 1 for which the linear fitting cannot derive an accurate *ν* index.

In Figure 13, we show the histogram of *γ* as derived by the data-analysis of the Wind observations over 2002, before (grey) and after the filter application with (green) α = 1, and (blue) α = 0.1. As expected, the filter selects a percentage of the available data-points. The filtered *γ* is recorded over a shorter range of values. The amount of filtered data-points decreases with decreasing α, which sets the accuracy requirements. There is a sharp dip in the histogram of the filtered values for *γ* = 1, which corresponds to isothermal plasma. For these intervals, the linear fitting cannot determine a value of the polytropic index *ν,* which is approaching ∞, thus, are excluded from the analysis. Finally, we note that here we apply the proposed filter in subintervals of fixed length (~8 min) in order to automatically validate the identity in Equation (8) with fixed accuracy. The plasma within the rejected subintervals is possibly measured with high uncertainty, and the accuracy requirement set by the filter is not met. Moreover, the plasma in those subintervals may not correspond in uniform plasma with a single polytropic index. More sophisticated methods can investigate the polytropic relation in different timescales and by applying more complicated polytropic models [3,4].

## 7. Summary and Conclusions

In this paper, we examined the misestimation of the space plasma polytropic index using spacecraft measurements and a linear fit to the measured ln*T* as a function of ln*n*. We quantify the expected error as a function of the plasma density and temperature measurement uncertainties, considering several characteristic polytropic behaviors. We further analyzed Wind observations in order to derive the polytropic index of the solar wind protons over 2002 and examined its behavior as a function of the measurement uncertainties.

We concluded that:

- Density measurement uncertainties shift the estimated polytropic index towards 1. For density uncertainties comparable with the range of density variation during the analyzed intervals, the linear fit cannot resolve any real expansion or compression of the plasma and returns artificial isothermal relation;
- Temperature measurement uncertainties introduce statistical error in the calculation of the polytropic index;
- The correlation coefficient drops drastically as the plasma measurement uncertainties increase, therefore, it can be used as a potential criterion to filter erroneous data. Nevertheless, such a criterion will also exclude real, nearly isothermal cases;
- When using standard least square fitting, we can filter erroneous data by calculating the special polytropic index *ν* and exclude intervals for which *ν* differs significantly from its definition value ${\left(\gamma -1\right)}^{-1}$. We demonstrated our suggested approach using the solar wind proton measurements by Wind in 2002.

Therefore, future analyses on the polytropic index determination must be cautious of the possible misinterpretations due to measurement uncertainties and apply appropriate filters to the data, as suggested here. Finally, we highlight the importance of the accurate determination of *γ* since it is a crucial parameter in understanding the physical mechanisms in plasmas and it is related to the kappa index that labels and governs the velocity distribution functions of the plasma particles [14,15,16]. Future studies can address the polytropic behavior of different solar wind structures, in different time scales, and by adjusting several data-selection criteria.
